# The adoption of conservation practices in the Corn Belt: the role of one formal farmer network, Practical Farmers of Iowa

**DOI:** 10.1007/s10460-023-10451-5

**Published:** 2023-05-03

**Authors:** L. Asprooth, M. Norton, R. Galt

**Affiliations:** 1 Geography Graduate Group, University of California, 129 Hunt Hall, One Shields Avenue, Davis, CA 95616 USA; 2 Bioeconomy Institute, Iowa State University, 1140 Biorenewables Research Lab, 617 Bissel Road, Ames, IA 50011 USA; 3 Department of Human Ecology, University of California, 1309 Hart Hall, One Shields Avenue, Davis, CA 95616 USA; 4 Agricultural Sustainability Institute, University of California, 143 Robbins Hall, One Shields Avenue, Davis, CA 95616 USA

**Keywords:** Peer-to-peer networks, Formal farmer networks, Adoption, Agriculture, Conservation practices, Corn Belt

## Abstract

Substantial evidence has shown that involvement in peer-to-peer farming networks influences whether a farmer decides to try a new practice. Formally organized farmer networks are emerging as a unique entity that blend the benefits of decentralized exchange of farmer knowledge within the structure of an organization providing a variety of sources of information and forms of engagement. We define formal farmer networks as farmer networks with a distinct membership and organizational structure, leadership that includes farmers, and an emphasis on peer-to-peer learning. This study complements existing ethnographic research on the benefits of organized farmer networking by examining farmers in one longstanding formal farmer network, Practical Farmers of Iowa. Using a nested, mixed-method research design, we analyzed survey and interview data to understand how participation and forms of engagement in the network are associated with the adoption of conservation practices. Responses from 677 farmers from a regular member survey disseminated by Practical Farmers of Iowa in 2013, 2017, and 2020 were pooled and analyzed. GLM binomial and ordered logistic regression results indicate that greater participation in the network, particularly through in-person formats, has a strong and significant association with greater adoption of conservation practices. Logistic regression results show that building relationships in the network is the most important variable for predicting whether a farmer reported adopting conservation practices as a result of participation in PFI. In-depth interviews with 26 surveyed member farmers revealed that PFI supports farmers to adopt by providing information, resources, encouragement, confidence building, and reinforcement. In-person learning formats were more important to farmers relative to independent formats because they were able to have side conversations with other farmers, ask questions, and observe results. We conclude that formal networks are a promising way to expand the use of conservation practices, particularly through targeted efforts to increase relationship building in the network through face-to-face learning opportunities.

Agriculture in the Corn Belt is dominated by industrial farming practices that have resulted in serious damages to the health of farmers, the environment, and rural communities. Regenerative agricultural practices that increase cropping diversity while using fewer chemical inputs and conserving soil and water resources are proven to be equally as productive as conventional systems while delivering greater economic and environmental benefits (Cruse et al. 2010; Davis et al. 2012; Dainese et al. 2019; Hunt et al. 2020; Tamburini et al. 2020). Despite these benefits, conservation practices are still relatively rare in the Midwest. In Iowa, the US state that produces the most corn, hogs, and eggs (Thessen et al. 2020), only 11% of cropland operations use cover crops, and 34% use conservation tillage, two critical conservation practices (USDA NASS 2017).

A farmers’ decision to adopt a conservation practice is influenced by a complex and interconnected set of social, economic, geographical, environmental, and agroecological factors which have been documented in a large empirical literature (Prokopy et al. 2008, 2019; Baumgart-Getz et al. 2012; Carlisle 2016). Of these factors, how and through whom information about a conservation practice reaches a farmer can impact whether they choose to adopt. Diffusion of innovation theory (Rogers 2003) and social network theory (Prell 2012) propose that an individual’s social network is central to the diffusion of new ideas and practices. Information about innovations is spread through social networks and the presence of specific individuals or actors within one’s network, and the information flowing through it, provide additional explanation as to whether an individual adopts a new practice (Wellman 1983; Valente 1996; Rogers 2003).

Within an individual’s network, connections with peers are particularly consequential to whether one adopts a new practice. Explained through the theory of social learning, or transmitting knowledge from peer-to-peer through interpersonal communication, individuals learn best by observing and replicating the behavior of others (Bandura 1977). Information diffused through interpersonal channels allows for the individual to observe and validate results, and to ask questions to decrease uncertainty about the practice (Ban 1981; Rogers 2003). As a result, an individual is more likely to adopt a new practice when information about the practice comes from someone they know and trust and who may have already tested the practice (Foster and Rosenzweig 1995; Rogers 2003; Granovetter 2005). These theories are supported by a large literature showing that farmers who are better connected to other adopters and grassroots organizations promoting adoption are more likely to adopt new practices (Prokopy et al. 2008; Baumgart–Getz et al. 2012; Carlisle 2016).

In addition to social learning, farmer networks provide mutual support and motivation to go against established norms in agriculture (Bell 2004; Kroma 2006; Gosnell et al. 2019). More broadly, participating in a social network can instill social pressure to conform to the norms of the group (Ajzen 1991; Montgomery and Casterline 1996; Hogset and Barrett 2010). Social networks of farmers are particularly important for diffusing information on alternative agricultural practices that may not be available through traditional research and extension (Kroma 2006), and for sharing local knowledge about conservation practices that are more information-intensive and site-specific compared to simplified industrial practices (Hassanein and Kloppenburg 1995; Lyon et al. 2011; Iles and Marsh 2012).

Social networks in agriculture can take many forms and much existing research has focused on personal communication networks of farmers through social network analysis (Garbach and Morgan 2017; Levy and Lubell 2018) and ethnographies of networks (Hassanein 1999; Warner 2007; Wypler 2019). Fewer studies have examined organized or formalized farmer-to-farmer networks, which we identify as a unique construct. In this paper, we look specifically at formal farmer networks to understand the significance of this type of peer organization in catalyzing farmers to adopt conservation practices in the Corn Belt.

## Formal farmer networks

A handful of studies have examined the role of formal agricultural networks in the context of social change in agriculture (Bell 2004; Trauger 2009; Barbercheck et al. 2014; Pape and Prokopy 2017; Šūmane et al. 2018). Within these studies, no shared definition of what constitutes a formal farmer network exists and only two differentiate between types of farmer networks. Pape and Prokopy (2017) define formal and informal social networks broadly where formal networks are structured organizations with defined roles and purposes (e.g., a club, church body, or team of coworkers) and informal networks are unstructured interrelations between individuals acting on their own terms, without centralized organization or planning (e.g., family, neighbors, and friends). Šūmane et al. (2018) specify formal knowledge networks in agriculture as formal institutions with a structured agenda, that receive public funding, and that circulate formal knowledge (e.g., universities, advisory services) as opposed to informal knowledge. Informal knowledge networks in agriculture are “fuzzier” relations between community members, family and personal relations that occur as part of a farmers’ daily routine (Šūmane et al. 2018, 237). Across these definitions, organizations are considered formal, while exchanges with personal relations are considered informal.

Yet, neither capture the characteristics of formalized peer-to-peer farmer networks, emerging as a unique entity that blends the benefits of the decentralized exchange of farmer knowledge within a structured organization. We briefly examined farmer organizations and associations in the US and found formal farmer-to-farmer networks to be a particular type, with the following shared characteristics: (1) a defined membership and organizational structure (2) leadership (e.g., board of directors) that includes farmers and (3) an emphasis on peer-to-peer learning. Of the organizations we examined, nine met these criteria including: Grassworks, Iowa Organics Association (IOA), Nebraska Sustainable Agriculture Society, Northeast Organic Farming Association (NOFA), Ohio Ecological Food and Farming Association (OEFFA), PASA Sustainable Agriculture, Practical Farmers of Iowa, Southeastern African American Farmers’ Organic Network (SAAFON), Sustainable Farming Association of Minnesota, and most producer-led watershed protection groups.

Across all formal farmer networks we examined, membership is state or regionally based and representative of various farming enterprises. Some of the networks (IOA, NOFA, OEFFA, SAAFON), have current or historic ties to organic-specific production. All of the networks have been established for over thirty years with the exception of IOA and SAAFON, both founded in 2006. The longest standing organization is NOFA (1971), followed by OEFFA (1979), and PFI (1985). PFI has the most robust programming offered to members; however, all formal networks offer some combination of programming and resources that include annual conferences; field days; written educational resources; mentorship or apprenticeship opportunities; email listservs; and/or classifieds to find or sell equipment, livestock, or post positions. Formal networks take on varying degrees of policy advocacy. Some organizations are proactive in farm policy, such as IOA or OEFFA, and others remain apolitical, such as Grassworks. While PFI advocates for policies promoting working lands conservation, other core areas of programming such as farmer-to-farmer learning events, cost-share, and on-farm research receive priority over direct policy work (Personal communication with PFI Marketing and Communications Director, December 2021).

While farmer cooperatives often result in the sharing of information and comradery inherent in networks, we do not consider them to be formal farmer networks as we define them, since cooperatives’ objectives are largely economic—e.g., to improve bargaining power (Schram 2010)—as opposed to social. While we attempt to characterize formal farmer-to-farmer networks, we recognize that networks are diverse and suited to the needs and resources of the community the network serves (White 2021).

Formal farmer networks are particularly important in the transition to sustainable agriculture because of their role in bridging two valuable forms of knowledge: farmers’ experiential knowledge shared between peers and formal scientific knowledge shared from professionally trained experts. Before the onset of public agricultural science, new knowledge was created through the cumulative experience of farmers and exchanged among themselves. This local knowledge has been largely devalued by the scientific community and loose networks of farmers sharing information have traditionally operated separately from agricultural extension (Kloppenburg 1991; Hassanein 1999; Kroma 2006). Agricultural extension primarily uses a top-down approach to disseminating scientific information from traditional “experts” to farmers (Warner 2007; Lubell et al. 2014). Criticisms of traditional agricultural extension have led to alternative research and extension models that involve the participation of farmers and other stakeholders (Chiffoleau and Desclaux 2006; Warner 2007; Healy and Dawson 2019) and the use of existing grower networks to extend agricultural science (Hoffman et al. 2015).

Still, these methods place the scientist and the extension professional at the center of the production of and dissemination of knowledge in agriculture, while research indicates that farmers are more likely to test or adopt innovations when information about the innovation is transmitted through peers (Rogers 2003). Formal famer networks are a promising collaborative way to produce and share both scientific and farmer knowledge because they privilege farmers’ knowledge and learning in the process of dissemination.

In formal farmer networks, membership fees, grants, and/or donations help support network programming. Leadership facilitates the sharing of information by linking farmers with each other and by linking farmers with outside actors and institutions. Farmers connect through newsletters, email discussion lists and social media, and through social learning activities such as on-farm research trials and field days. In many of these formats, farmers are in the teaching role and farmer-generated knowledge is shared between participants. Leadership ties in university researchers and extension staff, conservation professionals, and other agriculture specialists to the network through events such as webinars and workshops, often conducted alongside farmer presenters. Network leadership may provide technical assistance, navigate cost share opportunities, work with farmers to trial new practices, analyze data from field trials, produce reports, and distribute informational material members can access independently. The result is a diversity of both sources of expertise and ways of learning within the network that includes farmer and “expert” generated knowledge, and in-person and independent learning pathways.

Using Rogers’ (2003) explanation of diffusion, formal networks can be considered a hybrid of centralized and decentralized information exchange. In a centralized diffusion system, the decision to begin to diffuse an innovation is made by formal experts and spread downwards. In a decentralized system, information, often developed from practical experience, is spread horizontally among potential adopters. Formal farmer networks form a synergy between the benefits of peer exchange and access to information produced in institutions that may be missing from informal peer networks. With ties to both institutional actors and a network of farmers, formal farmer networks bridge social and institutional knowledge, playing a “boundary spanning” role (Levy and Lubell 2018).

In addition to experts sharing information with farmers, and farmers sharing information with each other, formal farmer networks provide opportunities for farmers to share feedback and information to experts, completing a feedback loop. In some formal farmer networks, farmers share their knowledge with agricultural researchers and provide feedback to agricultural scientists and extension agents on the performance of different conservation and production practices at events, on email discussion lists, and through the dissemination of research reports. Farmers also provide feedback to network leadership which drives programming decisions and signals to how to better assist members.

Despite the unique attributes of formal farming networks, few studies have quantitatively examined the role of participation in a formally organized peer-to-peer network in the adoption of conservation practices in the US. Within the body of literature predicting the adoption of best management or conservation practices, some studies include the degree of participation in community organizations (Belknap and Saupe 1988) and in agricultural or conservation organizations broadly (Korsching et al. 1983; Bates and Arbuckle 2017), which are found to be positively associated with adoption. Others examine the degree of farmer-to-farmer networking (Wilson et al. 2014), perceived effectiveness of learning from others including neighbors (Dunn et al. 2016), and importance placed on neighbors as an information source (Thomas et al. 1990), with varying results.

The two studies that consider a form of formal peer networks in quantitative studies of adoption are by Barbercheck et al. (2014) and Pape and Prokopy (2017). Barbercheck et al. (2014) looked at participation in various agricultural organizations among female farmers in the Northeastern US and the use of conservation practices. They found participation in an organized women’s farming network to be positively associated with the adoption of one of 11 measured cropping system conservation practices—manure incorporation after application (Barbercheck et al. 2014). Pape and Prokopy (2017) found that farmers in two formal networks—defined as structured organizations with defined roles and purposes—in Indiana use a range of conservation practices more than non-network farmers, most of the differences significant at the 0.1% level. They also found a positive and significant relationship between the length of time a farmer participated in a network and self-reported improvements to their nutrient management practices (Pape and Prokopy 2017). Our research adds to the limited body of evaluative evidence of the role of farmer networks in the US in two key ways: 1) we focus on formally organized farmer networks, complementing existing ethnographic research on their role in farmer decision making and 2) we measure the intensity of participation for a more nuanced understanding of the role of the network.

## Study context

We studied farmers within one longstanding formal farming network in the Midwest, Practical Farmers of Iowa (PFI), to understand how participation in the network relates to the adoption of conservation practices. PFI uses “farmer-led investigation and information sharing to help farmers practice an agriculture that benefits both the land and people” (PFI 2022a). The group has a history in the region of facilitating open dialogue between farmers to develop solutions to make farming more sustainable (Bell 2004). Established in 1985, a time when farmers were under great economic pressure, PFI was formed by a group of like-minded farmers who came together to increase diversity on their farms while also reducing input costs. Members learned from one another and organized to conduct randomized, replicated on-farm research to improve their profitability, efficiency, and stewardship (PFI 2022a). PFI has grown to an organization with over 6000 members and 30 staff with program areas that extend beyond the initial row crop and livestock enterprises to include horticulture, beginning farmers, habitat restoration, and policy. From the summer of 2019 to the spring of 2020, PFI’s events and workshops had 2750 total attendees (Personal communication with PFI Membership Manager, July 2021). Its members are primarily located in Iowa but include members across the country and internationally.

PFI member farmers, including both row crop and other types of member farmers, are different from the broader farming population in the region in that, on average, they are younger, have larger farms, and have lower rates of land ownership (USDA NASS 2017; PFI 2020). Member farmers have greater-than-average adoption of conservation practices compared to other farmers in the region. For example, only 11% of farmers across the Corn Belt reported using cover crops according to the most recent USDA agricultural census (USDA NASS 2017). Yet, in 2020, 67% of all PFI member farmers report using cover crops (PFI 2020). Similarly, PFI farmers report a higher use of reduced or no-till practices and enrollment in government conservation programs compared to the larger farming population in the region (USDA NASS 2017; PFI 2020).

## Research questions and hypotheses

Using data from the three most recent PFI member surveys in 2013, 2017, and 2020, and interviews with member farmers, we ask the following questions: (1) What is the role of the PFI farming network in the adoption of conservation practices amongst its members? (2) What drives successful participation in the network? and (3) Do the various ways member farmers participate and learn within the network matter for the adoption of conservation practices?

Knowing that the frequency and depth to which an individual interacts with a network increases the likelihood of the diffusion of information among participants (Cheng 2021), we measure participation for our first question by the degree of involvement in PFI events and workshops and engagement with PFI materials. The second question measures successful participation in the network by whether a farmer reported adopting a conservation practice as a result of participation in PFI. For our third question, we test whether in-person ways of participating traditionally associated with social learning in agriculture such as field days, socials, and workshops have a stronger relationship with adoption compared to independent ways of participation within the network more unique to formal farmer networks such as email discussion lists, participatory research reports, webinars, and podcasts.

Given the unique attributes of formal farmer networks to produce and share both scientific and farmer knowledge, and to provide opportunities for both social and independent learning, we hypothesize that participation in the network will be positively associated with the adoption of conservation practices. We theorize that engaging in a formal farmer network provides impactful opportunities to learn about conservation practices from other farmers and agricultural professionals while receiving support and validation, thereby increasing the likelihood one will adopt.

We also hypothesize that in-person and independent ways of engaging in the network do not have the same relationship with adoption. PFI offers a range of in-person events and ways of engaging remotely and accessing materials independently. The learning pathways a farmer can access independently within the PFI network can still be considered a blend of social and independent learning since they are created by or use data or information from other farmers. Nonetheless, we make a distinction between in-person participation and independent participation, hypothesizing that face-to-face interactions are more important for adoption than independent ways of learning and engaging within the network.

Existing theory of social learning and the diffusion of innovation holds that face-to-face, interpersonal channels of communication are more likely to influence an individual’s decision making compared to impersonal sources due to the ability to observe and ask questions about practices (Bandura 1977; Ban 1981; Rogers 2003). This type of learning is especially important for conservation practices that tend to be more complex and knowledge-intensive compared to conventional practices (Carlisle et al. 2019; Laforge and Levkoe 2021). Moreover, empirical literature in agriculture shows that farmers place more value on social learning compared to institutional or top-down learning (Hoffman et al. 2015; Laforge and McLachlan 2018) and that social learning (Garbach and Morgan 2017; Singh et al. 2018), and particularly face-to-face ways of receiving information (Bates and Arbuckle 2017), have been shown to be a strong predictor of the adoption of conservation practices.

Results from this study can provide insights to other organized farmer networks in the region, inform the programming and allocation of resources amongst agriculture professionals and policy makers, and inform academics and extension professionals of the role of organized networks among the many factors determining whether a farmer chooses to use a conservation practice.

## Methods and data

### Farmer survey

The quantitative analysis used data collected by PFI in 2013, 2017, and 2020 through their member survey, a survey distributed to all members every three to four years. Access to this data was given to the researchers by the staff of PFI, with identifying information removed. The member surveys were disseminated via email and collected information about members’ farms, farming practices, goals, feedback about past PFI programming, and future priorities for PFI. Members were encouraged to complete the survey through an online form, but were also given the option to complete a paper copy. The 2020 survey was disseminated in December of 2019 and responses were collected until March, 2020; the 2017 survey was disseminated February of 2017 and responses collected until July; and the 2013 survey was disseminated in September of 2013 and responses were collected until January of 2014. Over the course of each campaign, members were sent between one to four email reminders and staff posted general reminders in the e-newsletter and on the email discussion list. Incentives were offered to take the survey in the form of drawings for PFI promotional materials.

In 2020, 785 of 1941 member households responded for a response rate of 40%, in 2017, 650 of 1431 member households responded for a response rate of 45%; and in 2013, 660 of 1286 member households responded for a response rate of 51%. Ninety-five percent of surveys in 2020 were collected before the onset of the Covid-19 pandemic as of March 11, 2020. Thus, the 2020 survey does not likely reflect changes in participation due to the pandemic. Cross sectional data from the three different survey periods were pooled and the most recent survey was used for individuals that completed the survey multiple years.

Only members who reported that they were currently farming were included in the analysis. We did not include other types of PFI members, such as farmland owners who do not operate their land, aspiring farmers, or friends of farmers. Farmers cultivating corn and/or soybeans, among other crops, are the largest membership contingency within PFI and constitute 53% of the farmers who responded to the membership survey. The other 47% of respondents cultivated a range of vegetables, fruits, flowers, hay, and livestock. We excluded farmers who had only been a PFI member for one year or less at the time of the survey, since we assume that it was less likely that PFI would have been the driver of their use of conservation practices. We judge a time lag of one year or greater to be a sufficient amount of time to adopt the majority of the practices measured.

After exclusions, the final sample size of the pooled cross-sectional data was 677 member farmers including 118 farmers in 2013, 149 in 2017, and 410 in 2020. The network attracts members throughout the US, although most live in Iowa and surrounding Corn Belt states. The states represented by farmers in the sample include Alabama (1), Arizona (1), California (1), Colorado (1), Iowa (590), Illinois (15), Indiana (1), Kansas (2), Kentucky (1), Michigan (1), Minnesota (23), Missouri (4), Montana (1), Nebraska (11), New Jersey (1), Ohio (6), Ontario Canada (1), Pennsylvania (1), South Dakota (5), and Wisconsin (10). Given that the vast majority of farmers in the sample reside in Corn Belt states, Iowa in particular, we use this region to compare our sample.

### Regression variables

**Fig. 1:**
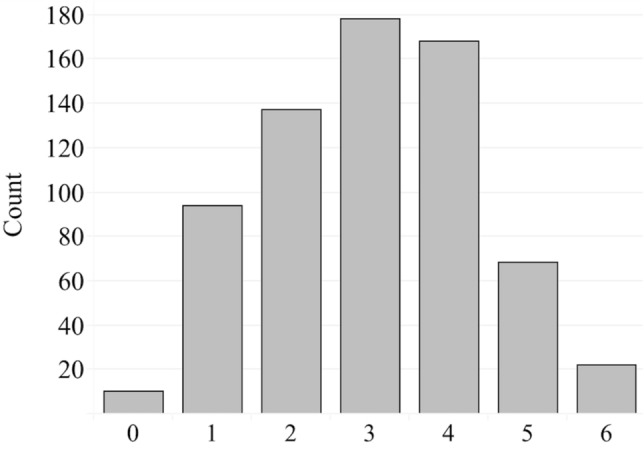
Distribution of the dependent variable *adoption of conservation practices index* using the pooled member survey data from 2013, 2017, 2020 (*n* = 677)

The first dependent variable, the *adoption of conservation practices index*, is a composite index ranging from 0 to 6 based on the sum of farmer-indicated use of the following practices (each are binary variables coded as yes = 1 and no = 0): buffer or filter strips (riparian, grass, prairie), conservation tillage (no till, strip-till, ridge-till, or reduced till), cover crops, flame or electric weeding, low-use of synthetic inputs (organic farming or no use of synthetic inputs), and sustainable grazing (rotational grazing or high-density/adaptive multi-paddock grazing). A score of 6 indicates use of all conservation practices listed and a score of 0 indicates no use of the practices listed. The distribution of the *adoption of conservation practices* index is displayed in Fig. 1.

These practices were derived from the member survey and are key practices elevated by PFI, clearly improving of conservation on farms, and applicable to all farms in the sample. While conservation practices have different levels of environmental and agronomic benefit (Reimer et al. 2014), we do not weight each practice within the adoption index. A lack of comparable data on the relative environmental impact across each practice, and the varying impact depending upon the biophysical conditions of each farm which are spread across a heterogenous geographical area, would make it difficult to do so. While some studies have attempted to differentially weight certain practices (Napier and Tucker 2001; Napier and Bridges 2002), other studies have used composite indexes similar to ours when analyzing adoption of best management practices in agriculture (Thomas et al. 1990; Park and Lohr 2005; Filson et al. 2009; Campbell et al. 2011).

The independent variable *general participation index* measures a farmer’s participation in a range of events and resources offered through the network. We created an index according to the number of events attended and engagement with resources reported by the farmer completed in the past 12 months. In addition, we test the association of ways of participating and learning in the network and the adoption of conservation practices. We do so by defining two types of participating and learning—in-person and independent. In-person ways involve face-to-face social learning and independent ways allow the farmer to learn on their own through PFI materials.

We disaggregated the *general participation index* into two discrete indexes of in-person and independent ways of participation (Table 1). The *in-person participation index* includes the following categories: attended 1–2 events, attended 3 or more events, and acted as a farmer-leader. Acting as a farmer leader includes at least one of the following: conducted on-farm research, hosted a field day, talked to the media, served on a committee, or served as a mentor. This category is weighted doubly to account for greater integration into the network. The *independent participation index* includes: visited the PFI website, read the PFI weekly e-newsletter, and used the PFI e-mail discussion list. Each category that was checked is given a 1 (except for farmer-leader, which is weighted doubly as noted above) and summed for a final score, ranging from 0 to 7 for the combined index, 0–4 for the in-person index, and 0–3 for the independent index.

**Table 1:** Main independent variable composition

Included Variables	General participation index (0–7)	In-person participation index (0–4)	Independent participation Index (0–3)	Survey question
Attended 1–2 events	Y	Y		The following are events and resources offered through PFI. Please select any you have done in the past 12 months
Attended 3 or more events	Y	Y		
Acted as a farmer-leader (doubly weighted)	Y	Y		
Visited the PFI website	Y		Y	
Read the PFI weekly e-newsletter	Y		Y	
Used the PFI e-mail discussion list	Y		Y	

Each model includes additional covariates also shown to be associated with adoption in review studies (Baumgart-Getz et al. 2012; Carlisle 2016; Prokopy et al. 2019) and narrowed their inclusion to the best model fit according to those that increased the *R*^2^ value. Of the predictor variables gleaned from the member survey, *age of primary member* farmer, total *farm size* in acres, *participation in EQIP* (Environmental Quality Incentives Program), *participation in a watershed group*, *proportion of acres owned*, and *years in PFI*, were included in the model.

*Age of primary member* is the age of the person who completed the survey. *Farm size* is the combination of acres owned and rented that are operated by the member farmer. *Participation in EQIP* measures present or past participation in EQIP. *Participation in a watershed group* measures current or past involvement in a watershed project. Watershed projects are farmer-led, governmentally funded cost-share programs with the aim of improving water quality through the use of conservation practices. *Proportion of acres owned* measures the proportion of acres owned and operated to total acres operated (rented and owned) and ranges from 0 to 1. The number of years of membership in PFI (*years in PFI*) was included to control for increased adoption due to length of time in the network and to isolate the association of increased participation and the adoption of conservation practices. A dummy variable for year in which the survey was distributed was included as a fixed effect to control for variation across years such as fluctuations in government conservation program supports, commodity prices, and weather which may have impacted adoption. The state in which the farmer is located (*state*) was included to control for distance to in-person events, as over 95% of PFI events are held in the state of Iowa (Personal communication with PFI Membership Manager, July 2021). Including state fixed effects also help to control for time-invariant environmental characteristics at the state-level that may influence the adoption of conservation practices such as climate and soil type.

The second dependent variable is *adopted a conservation practice(s) as a result of participation in PFI*.^1^ We selected independent variables available through survey data to predict whether a farmer adopted conservation practice(s) as a result of participation in PFI, including *age of primary member*, whether the farmer reported having formed relationships through the network (*formed relationships*), whether the farmer reported having felt a sense of community through the network (*felt a sense of community)*, *years in PFI, state*, and year fixed effects.

### Regression analysis

Using R statistical software (Version 4.2.1), we created a series of regression models to understand the relationships between the *adoption of conservation practices index* and the three key independent variables described in Table 1: general, in-person, and independent participation in PFI. The three main independent variables were included in separate models due to theoretical and observed issues of multicollinearity. For general participation, we ran bivariate regressions between the *adoption of conservation practices index* and the *general participation index*, a multivariate regression with covariates and fixed effects, and a multivariate regression with covariates and fixed effects including an interaction between the *general participation index* and the number of *years in PFI.* The interaction was chosen due to the likelihood that general participation in PFI had a different relationship with adoption depending on the number of years the member had participated in the network. For in-person and independent modes of participation, we ran multivariate regressions with covariates and fixed effects. In addition, we ran a multivariate regression to understand the factors that predict whether a farmer adopted conservation practice(s) as a result of participation in PFI. We standardized the independent variables in all models to compare the relative importance of each while holding all other variables in each model constant.

We estimated the association with the *adoption of conservation practices index* and the independent variables in the model using two model types: a Generalized Linear Model (GLM) based on the binomial probability distribution and an ordered logistic model. The binomial GLM model best fits data with a dependent variable that is a discrete count composed of positive integers with an upper bound (McCullagh 2019). Unlike other count models, the binomial GLM model predicts the sum of the number of “successes” of a practice being adopted out of a set number of independent “trials” and will therefore not predict values outside the range of the adoption index. Because this data may have minor violations of independence of trials from the same individual, we conducted an ordered logistic regression to test the robustness of the binomial GLM results. For the ordinal logistic regression, we treated the dependent variable, *adoption of conservation practices index,* as ordered and discrete, allowing for the distances between intervals in the dependent variable to vary (Pampel 2021). A binary logistic regression model is used to predict the factors associated with the variable *adopted conservation practice(s) as a result of participation in PFI,* appropriate for models with a binary dependent variable.

Results for each model are expressed as the likelihood of being in a higher level of adoption with a unit increase in each independent variable (Models 1–6 and 8–11), or the likelihood of having adopted conservation practice(s) as a result of participation in PFI (Model 7) controlling for all other independent variables. We calculated a McFadden’s pseudo *R*^2^ to measure the predictive power of each model, an appropriate measure for the generalized linear, ordered logistic, and binary logistic regression with no *R*^2^ value. To verify that our models did not violate assumptions and to improve confidence in our model selection, we performed a series of examinations for each model type. A study of correlation coefficients showed no signs of multicollinearity among the independent variables in any of the models. Residual versus fitted scatter plots indicated that the relationships between the independent and dependent variables were linear and the variance of the error terms constant. For the ordinal logistic regressions, a test of parallel lines (Brant 1990) showed no indication of a varied effect of the independent variables across levels of the adoption index, except for the variables farm size and year = 2013. Given that proportional odds held for the key independent variables across the fitted models, we believe that the dependent variables are more appropriately treated as ordinal compared to nominal. No evidence of lack of model fit was found using a Wald Chi-squared test across all models, nor for the ordinal logistic models using a Lipsitz, Hosmer–Lemeshow and Pulkstenis-Robinson tests (Fagerland and Hosmer 2016).

### In-depth interviews

In-depth, semi-structured interviews with a subset of the 410 farmers who filled out a PFI member survey in 2019–2020 took place from July to December of 2020. Nesting our research design, we sampled farmers to interview from the member survey using a stratified, maximum variation sampling technique (Patton 2002) to understand perspectives from a variety of member farmers. First, we limited the sample to those growing corn and or soybeans to focus on the group of farmers with the greatest environmental impact on the Midwestern landscape and representative of a majority of PFI’s membership. Next, we divided surveyed farmers into four groups based on two axes, (1) their score on the adoption of conservation practices index and (2) their score on the general participation index, and randomly sampled within each of these four groups. Finally, after finding our sample to be composed of primarily men, we recruited and interviewed two additional women farmers through snowball sampling. Thirty-five farmers were contacted to participate in an interview; 71% agreed and were interviewed. In total, we interviewed 22^2^ current members and 4 previous member farmers who had not renewed their membership. The farmers we interviewed represent 1.3% of member households in the PFI network.

Farmers were told in the beginning of the interview that PFI was financially supporting the study, but that the interviewers were independent and no information from the interviews would be published or relayed back to PFI staff without all individual identifying information removed. Interviews were conducted via Zoom or in person depending on location and farmer preference, and lasted between 45 and 90 min. Questions focused on the factors that allowed them to transition to conservation practices, preferred learning and information sources, and experience with and opinions on the PFI network.^3^ Interview data were analyzed using NVivo software and coded according to an inductive approach that identified reoccurring themes.

## Results

### PFI and the adoption of conservation practices

#### Regression results

**Fig. 2:**
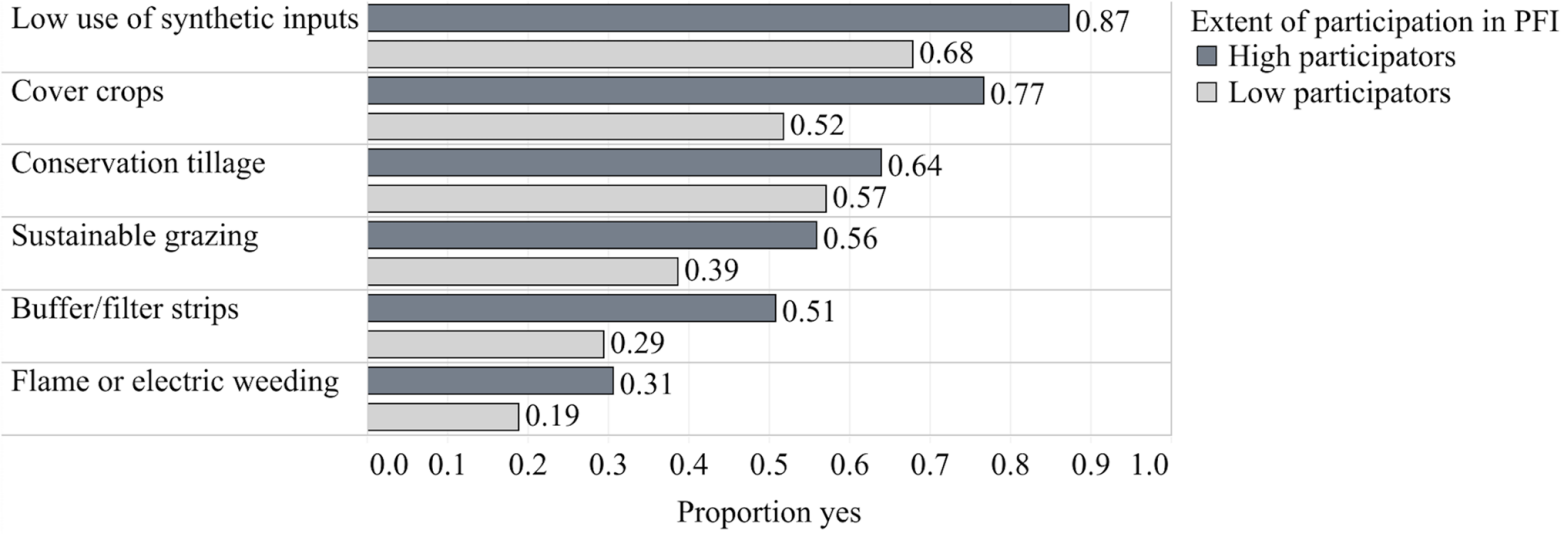
Dependent variable *adoption of conservation practices index* disaggregated by practice and by proportion that selected yes using the pooled member survey data from 2013, 2017, 2020 (*n* = 677) and further disaggregated by level of participation in PFI (High: *n* = 292; Low: *n* = 385)

The dependent variable, *adoption of conservation practices index*, is displayed disaggregated by practice and by the extent of participation in the PFI network in Fig. 2. Each practice that was included in the index is displayed by the proportion of member farmers that reported use. Low use of synthetic inputs and use of cover crops had the highest adoption among PFI farmers, at 75% and 61%, respectively. To understand how the adoption of each practice varied by the extent of participation in the network, we divided farmers into two groups, high and low participators, where high (*n* = 292) participators engaged in 4–7 of the activities listed and low participators (*n* = 385) engaged in 0–3. For all conservation practices, high participators are also those with higher rates of adoption, on average (Fig. 2).

**Fig. 3:**
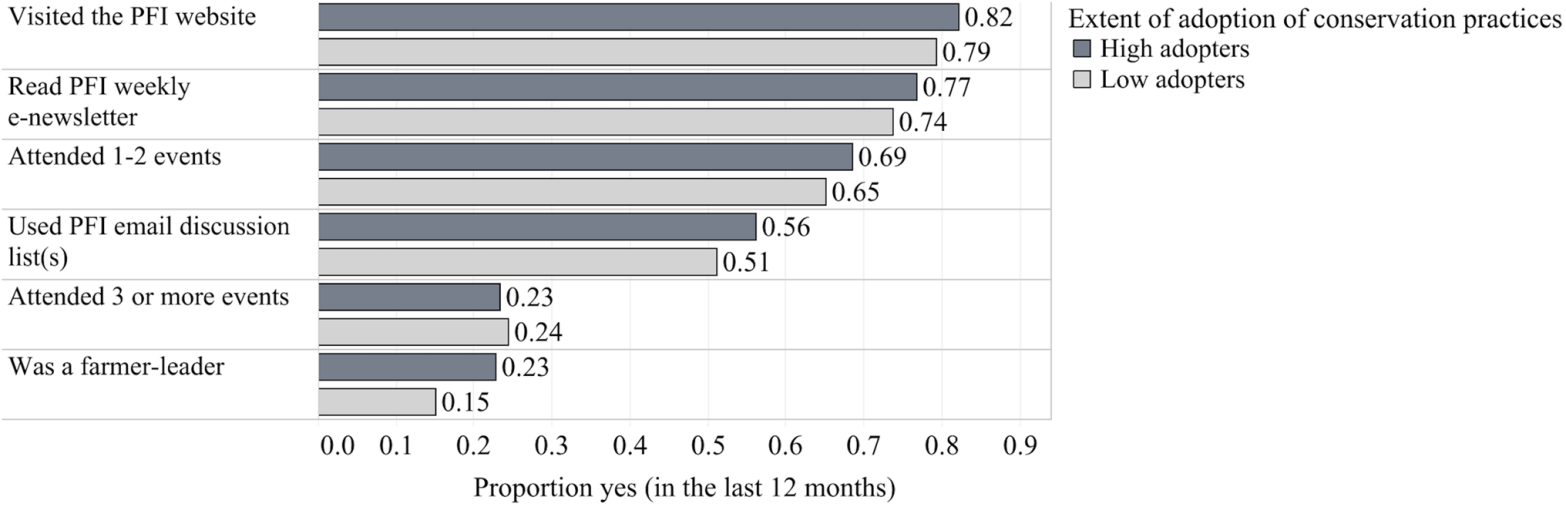
Main independent variable *general participation index* disaggregated by activity/resource and by proportion that selected yes using the pooled member survey data from 2013, 2017, 2020 (n = 677) and further disaggregated by level of adoption of conservation practices (High: *n* = 258; Low *n* = 419)

Figure 3 shows our main independent variable, *general participation index*, disaggregated by each type of activity or resource and by level of adoption of conservation practices. The most common way PFI member farmers participated in the network was through the PFI website. Over 80% of farmers said that they visited the website in the past 12 months. The PFI website includes resources organized by program area, PFI publications including the quarterly magazine, annual report, the Cooperators’ Program on-farm research reports, a member portal, and information about upcoming events. Farmers also frequently reported reading the PFI weekly e-newsletter (75%), attending 1–2 events per year (66%), and using the PFI email discussion list (53%). The newsletter shares information about upcoming events, member and organizational achievements or features in the media, publications, announcements, and highlights of timely resources. PFI offers in-person events including field days, conferences, workshops, regional meet-ups, socials, multi-day bus trips, and mentorship opportunities. Discussion lists are used by members and staff to ask and answer questions, post items for sale or needed, send announcements, and disseminate information on other resources and events outside of PFI. Specific lists exist concerning general announcements, field crops, livestock, and horticulture, and a perspectives list that provides space for members to engage in open-minded debate and discussion related to agriculture (PFI 2022b). Fewer farmers attended 3 or more events per year, or served as a farmer leader (i.e., conducted on-farm research, hosted a field day, talked to the media, served on a committee, or served as a mentor). Figure 3 also displays participation in the network by level of adoption of conservation practices where high adopters (*n* = 258) used between 4 and 6 conservation practices and low adopters (*n* = 419) used between 0 and 3. High adopters participated slightly more in each avenue listed besides attending 3 or more events, which was almost equal across adopter groups.

Table 2 summarizes the results of the regressions measuring the relationship between the *adoption of conservation practices index* and the *general participation index*. The results show that farmers’ level of participation in PFI is statistically significant in explaining the level of adoption of conservation practices. The higher a farmer's level of participation in PFI, the more likely it is that they will have a higher level of adoption of conservation practices, on average. Results are robust across both the bivariate and multivariate GLM binomial (Models 1 & 2) and ordinal logistic (Models 4 & 5) models. General participation in PFI has the second most statistically significant relationship with adoption, following participation in EQIP, across all covariates (excluding Year 2013 fixed effects) included in the GLM binomial Model 2 (p = 0.0001) and ordinal logistic Model 4 (p = 0.0003). These findings support existing scholarship showing that the degree of participation in conservation organizations (Korsching et al. 1983; Belknap and Saupe 1988; Bates and Arbuckle 2017) and the degree integration into or centrality within social networks broadly (Phelps et al. 2012; Cheng 2021) drive the diffusion of technology.

**Table 2:** GLM binomial and ordinal logistic regression models predicting adoption of conservation practices using the pooled member survey data from 2013, 2017, 2020

	Dependent variable:					
	Adoption of conservation practices index					
	GLM logistic			Ordered logistic		
	Model (1)	Model (2)	Model (3)	Model (4)	Model (5)	Model (6)
General participation index	0.121^***^	0.148^***^	0.123^***^	0.220^***^	0.298^***^	0.251^***^
	(0.032)	(0.038)	(0.039)	(0.068)	(0.081)	(0.083)
	p = 0.0002	p = 0.0001	p = 0.002	p = 0.002	p = 0.0003	p = 0.003
Age of primary member		0.015	0.007		0.011	− 0.007
		(0.042)	(0.043)		(0.092)	(0.092)
		p = 0.731	p = 0.873		p = 0.904	p = 0.942
Farm size		0.087^**^	0.084^**^		0.150^*^	0.146
		(0.042)	(0.042)		(0.091)	(0.092)
		p = 0.038	p = 0.047		p = 0.098	p = 0.111
Participation in EQIP (yes = 1)		0.166^***^	0.170^***^		0.354^***^	0.359^***^
		(0.039)	(0.039)		(0.084)	(0.085)
		p = 0.00002	p = 0.00002		p = 0.00003	p = 0.00003
Participation in a watershed project (yes = 1)		0.055	0.045		0.109	0.094
		(0.036)	(0.037)		(0.075)	(0.076)
		p = 0.129	p = 0.218		p = 0.149	p = 0.217
Proportion of acres owned		− 0.120^***^	− 0.114^***^		− 0.246^***^	− 0.238^***^
		(0.040)	(0.040)		(0.087)	(0.087)
		p = 0.003	p = 0.005		p = 0.005	p = 0.007
Years in PFI		− 0.001	− 0.015		0.012	− 0.011
		(0.040)	(0.041)		(0.086)	(0.087)
		p = 0.981	p = 0.709		p = 0.893	p = 0.901
State		0.073^**^	0.070^**^		0.170^**^	0.162^**^
		(0.035)	(0.035)		(0.074)	(0.074)
		p = 0.034	p = 0.043		p = 0.022	p = 0.029
Year 2017		0.001	0.009		0.005	0.021
		(0.042)	(0.042)		(0.091)	(0.092)
		p = 0.986	p = 0.832		p = 0.961	p = 0.818
Year 2013		0.199^***^	0.195^***^		0.477^***^	0.466^***^
		(0.038)	(0.038)		(0.085)	(0.085)
		p = 0.00000	p = 0.00000		p = 0.00000	p = 0.00000
Combined participation index*			0.123^***^			0.226^***^
Years in PFI			(0.036)			(0.075)
			p = 0.001			p = 0.003
Constant	0.015	0.071^*^	0.056			
	(0.031)	(0.037)	(0.037)			
	p = 0.637	p = 0.056	p = 0.133			
Observations	677	516	516	677	516	516
Log Likelihood	− 1,158.620	-835.720	− 829.512			
Pseudo R^2^ (McFadden’s)	0.02	0.13	0.15	0.005	0.28	0.28
Akaike Inf. Crit	2,321.24	1,693.44	1,683.02	2,304.03	1,697.25	1,689.96
Wald Chi-squared	0.22	76.40***	82.90***	10.31**	80.56***	88.98***
Hosmer and Lemeshow test				p = 0.95	p = 0.73	p = 0.59
Lipsitz test				p = 0.58	p = 0.90	p = 0.49
Pulkstenis-Robinson test				p = 0.62	p = 0.58	p = 0.79

Asterisks and bolding note statistical significance at or below the 10% level; ^⋆^ is < 0.1; ^⋆⋆^ is < 0.05, and ^⋆⋆⋆^ is < 0.01

Standard errors are listed in parentheses. All coefficients have been standardized

**Fig.4:**
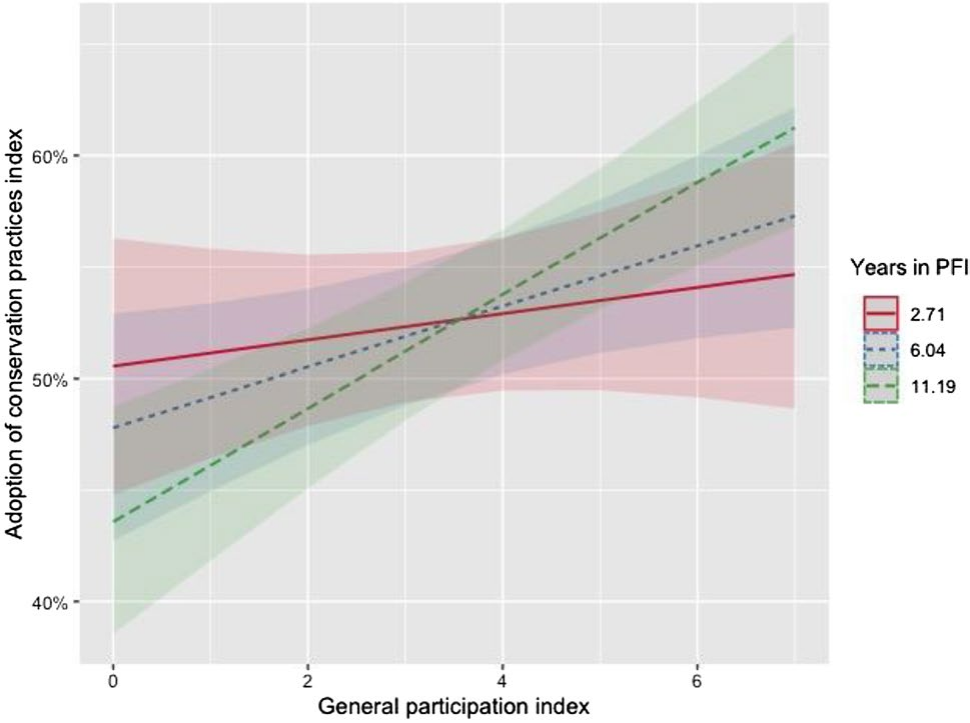
Interaction plot between the percentage of practices adopted within conservation practices index and the general participation index by the number of years in PFI (*n* = 677). The colors represent varying levels of years in PFI. Years in PFI are shown in the lower, median, and upper quartiles. The width of the bands of each group of years in PFI represent the upper and lower values of the 95% confidence intervals

While *years in PFI* is not significant in the multivariate Models 2 and 5*,* when interacted with the *general participation index*, the two combined have a strong effect on the *adoption of conservation practices index* (Model 3: p = 0.001, Model 6: p = 0.003). Figure 4 visually displays the moderating role of the length of time in the network on the relationship between participation and the adoption of conservation practices. For farmers who have been in the network for 11 years (long dotted green line), the association between their participation in PFI and their adoption of conservation practices is the strongest, indicated by the steepest slope. For farmers who have spent less than three years in the network (solid red line), the association between their participation in PFI and their adoption of conservation practices is the weakest, shown by the flattest slope. Thus, the interaction term in Models 3 and 6 and the relationships displayed in Fig. 4 show that the effect of greater participation in the network is the strongest when a member has been in the network for a longer period of time. Some existing research points to the importance of length of time in a farmer network to the adoption of conservation practices; Pape and Prokopy (2017) found that the length of time spent in formal networks in Indiana was positively associated with the use of better nutrient management practices. However, ours is the first research to date that has examined the combined effects of length of time spent in a farmer network and the degree of participation on the adoption of conservation practices.

Across the covariates, a farmers’ *participation in EQIP* and *proportion of acres owned* are associated with adoption at the 1% level of significance in all multivariate models (Models 2–3 & 5–6). The association between participation in EQIP and higher adoption is in line with several review studies that find conservation program participation has an overall positive relationship with adoption of conservation practices (Knowler and Bradshaw 2007; Prokopy et al. 2008; Carlisle 2016). The more acres of their total operation a PFI member farmer owns, as opposed to rents, the less likely they are to adopt a greater number of conservation practices was a surprising finding given that existing theory points to the advantages of owning land for receiving the longer-term benefits of conservation practices (Belknap and Saupe 1988; Soule et al. 2000). However, renting more acres may allow farmers to expand their farm size and larger farms are shown to enable farmers to absorb risk and adopt more due to economies of scale (Khanna et al. 1999; Ulrich-Schad et al. 2017; Durant et al. 2023) which may explain why farm size is also significant at the 5% level in the GLM multivariate models (Models 2 & 3). Within the fixed effects, we note that 2013 is strongly associated with adoption compared to 2017 or 2020. In the years following 2013, there was a greater effort placed on membership recruitment and a broader group of both conservation and conventional-minded farmers joined PFI (Personal communication with PFI Senior Programs and Member Engagement Director, November 2021). For this reason, it is likely that in 2013, adoption rates were higher due to the composition of the membership that tended towards higher adopters.

Knowing that participation in PFI is important for the adoption of conservation practices among members, it is useful to understand more precisely what drives successful participation in the network. Table 3 shows the logistic regression model (Model 7) with the dependent variable *adopted conservation practice(s) as a result of PFI* and several independent variables from the survey data that might predict the change. We find that, on average, those who said they *formed relationships* through the network were the most likely to report *adopted conservation practice(s) as a result of PFI* in terms of significance (*p* < 0.000), and coefficient size (1.789) holding all other variables in the model constant. This is in line with theories of social learning suggesting that farmers who are friends or in intentional relationships share more information, increasing the likelihood of adoption (Liverpool-Tasie and Winter-Nelson 2012; Mekonnen et al. 2022). Additionally, those who participated more (*general participation index*, p = 0.014) and those who reported feeling a sense of community (*felt a sense of community through the network*, p = 0.026) were also more likely to report adopting a conservation practice as a result of PFI. The *age of primary member*, the number of *years in PFI*, and the *state* in which they reside are not significant. Taken together, the independent variables in the model are strong predictors of whether a farmer adopted one or more conservation practices as a result of PFI indicated by a high pseudo R^2^ (0.55).

**Table 3:** Logistic regression model predicting the change of conservation practices as a result of participation in PFI using the pooled member survey data from 2013, 2017, 2020

	Dependent variable:
	Adopted conservation practice(s) as a result of participation in PFI
	Model (7)
General participation index	0.491^**^
	(0.198)
	p = 0.014
Age of primary member	0.012
	(0.173)
	p = 0.945
Formed relationships (yes = 1)	1.789^***^
	(0.150)
	p = 0.000
Felt a sense of community (yes = 1)	0.335^**^
	(0.150)
	p = 0.026
Years in PFI	0.179
	(0.191)
	p = 0.348
State	− 0.085
	(0.146)
	p = 0.562
Year 2017	− 0.328^**^
	(0.147)
	p = 0.027
Year 2013	− 0.583^*^
	(0.314)
	p = 0.064
Constant	1.386^***^
	(0.202)
	p = 0.000
Observations	548
Log Likelihood	− 145.904
Pseudo R^2^ (McFadden’s)	0.55
Akaike Inf. Crit	309.808
Wald Chi-squared	47.30***

Asterisks and bolding note statistical significance at or below the 10% level; ^⋆^ is < 0.1; ^⋆⋆^ is < 0.05, and ^⋆⋆⋆^ is < 0.01

Standard errors are listed in parentheses. All coefficients have been standardized

#### Farmer-reported results

Farmers reported their own experience with PFI and the use of conservation practices through the survey and through in-depth interviews. Using the pooled survey data from 2013, 2017, and 2020 we find that 71% of farmers said they adopted conservation practice(s) as a result of their participation in PFI (Table 4). Similar to the survey, when asked about the role of PFI in their transition to sustainable practices during in-depth interviews, 68% of farmers told us that their engagement in PFI helped. Farmers said that the PFI network provided information, resources, encouragement, and confidence building. Below we provide two illustrative examples from the 18 farmers who noted this. Farmer 2 explained:

I feel like PFI has been a huge source of information and encouragement…Probably, because of PFI's opportunities for learning and information sharing, it has given us maybe confidence to try something we might've been thinking about.

Farmer 3 echoed the regression findings on the importance of the length of time spent in the network for the impact of participation to be fully realized, which they attribute to the ability to observe practices used successfully by other members over longer periods of time:

I probably wouldn’t have used cover crops quite as much if I hadn’t seen PFI members doing it for a long period of time. That gave me confidence to try. Even though I probably philosophically knew it was the right thing to do, it was probably a little bit scary for me to do it at the beginning…seeing people do that… that were farmers, allows me to, not be quite as scary to try something like that.

Two other farmers noted that PFI helped to reinforce their decisions to adopt a new practice:

Farmer 16: I’m not sure I’ve changed a lot of practices with PFI…But they have solidified some things that, yes, I think I'm going down the right track. Or maybe I’ve learned something that’s tweaked the practice a little bit.

Farmer 19: The role of PFI in my life was to reinforce the decisions that I was making. To feel validated, I guess, about the decisions that I was making…So, I was already headed down the road toward a more diversified production, longer rotations, soil conservation, protecting the quality of the water. Heading down that road. But PFI just kind of gave me the validation and reinforcement that I felt like I needed to keep doing that. Which is an important role. I mean, you know?

**Table 4:** Famer reported experience of the PFI network using the pooled member survey data from 2013, 2017, 2020

	Pct	SD	*n*
Adopted conservation practices as result of participation in PFI (1 = Yes)	71%	0.45	667
Formed relationships through the network (1 = Yes)	73%	0.44	567
Felt a sense of community through the network (1 = Yes)	78%	0.41	570

Farmers also reported on the social impacts of their participation in the member survey. Seventy-three percent of farmers said that they formed friendships, business relationships, or relationships through the network and 78% percent reported feeling a sense of community through the network (Table 4). In an open-ended survey question about the impact of their PFI relationships, the most common responses included receiving new information and ideas, learning from the experience of others, and feeling social support from like-minded farmers. These farmer-reported impacts of network participation and the results of the regression in Table 3 show that the personal relationships formed through the network, together with greater participation (as measured by the *general participation index*) and a widespread sense of community, are important to explaining successful participation in the network.

### Ways of participating and learning in the network

#### Regression results

When considering ways of participating in the network separately, engagement through both in-person (Models 8 & 9) and independent (Models 10 & 11) pathways have a positive and significant relationship with increased adoption of conservation practices (Table 5). Thus, participating more, regardless of the format, is associated with greater adoption. However, in-person participation has a stronger relationship with adoption compared to independent participation in terms of statistical significance and the standardized coefficient size across both GLM binomial and ordinal logistic models. We use the McFadden’s Pseudo *R*^2^ and Akaike Information Criterion) (AIC) values to compare model fit across the in-person and independent participation models. The best model is signified by the highest Pseudo *R*^2^ value and lowest AIC value. A difference of 2 between the AIC values is considered substantial (Burnham 2002). The in-person participation models (Models 8 & 9) have higher Pseudo *R*^2^ values and AIC values are 10 units lower than the independent participation models (Models 10 & 11), indicating that in-person participation is significantly better than independent participation at predicting the level of adoption of conservation practices.

**Table 5:** GLM binomial and Ordinal logistic regression models comparing in-person and independent participation indices and the level of adoption of conservation practices using the pooled member survey data from 2013, 2017, 2020

	Dependent variable			
	Adoption of conservation practices index			
	GLM logistic	Ordered logistic	GLM logistic	Ordered logistic
	Model (8)	Model (9)	Model (10)	Model (11)
In-person participation index	0.124^***^	0.248^***^		
	(0.032)	(0.066)		
	p = 0.0001	p = 0.0002		
Independent participation index			0.087^**^	0.176^**^
			(0.041)	(0.088)
			p = 0.032	p = 0.046
Age of primary member	0.005	− 0.003	− 0.001	− 0.023
	(0.042)	(0.091)	(0.042)	(0.091)
	p = 0.911	p = 0.976	p = 0.981	p = 0.804
Farm size	0.083^**^	0.143	0.080^*^	0.136
	(0.041)	(0.088)	(0.042)	(0.092)
	p = 0.045	p = 0.104	p = 0.056	p = 0.137
Participation in EQIP (yes = 1)	0.168^***^	0.359^***^	0.177^***^	0.370^***^
	(0.038)	(0.084)	(0.038)	(0.084)
	p = 0.00002	p = 0.00002	p = 0.00001	p = 0.00002
Participation in a watershed project (yes = 1)	0.046	0.089	0.060	0.114
	(0.036)	(0.075)	(0.037)	(0.076)
	p = 0.203	p = 0.235	p = 0.101	p = 0.137
Proportion of acres owned	− 0.117^***^	− 0.242^***^	− 0.126^***^	− 0.252^***^
	(0.040)	(0.087)	(0.040)	(0.087)
	p = 0.004	p = 0.006	p = 0.002	p = 0.004
Years in PFI	0.007	0.025	0.015	0.038
	(0.040)	(0.086)	(0.040)	(0.086)
	p = 0.859	p = 0.773	p = 0.712	p = 0.662
State	0.077^**^	0.174^**^	0.059^*^	0.138^*^
	(0.035)	(0.074)	(0.034)	(0.073)
	p = 0.027	p = 0.019	p = 0.087	p = 0.058
Year 2017	0.008	0.020	-0.001	0.011
	(0.042)	(0.091)	(0.042)	(0.091)
	p = 0.849	p = 0.830	p = 0.978	p = 0.906
Year 2013	0.201^***^	0.483^***^	0.182^***^	0.447^***^
	(0.038)	(0.085)	(0.037)	(0.085)
	p = 0.00000	p = 0.00000	p = 0.00000	p = 0.00000
Constant	− 0.085		− 0.107	
	(0.055)		(0.093)	
	p = 0.127		p = 0.251	
Observations	516	516	516	516
Log Likelihood	− 835.653		− 841.043	
Pseudo R^2^ (McFadden’s)	0.13	0.28	0.11	0.27
Akaike Inf. Crit	1,693.31	1,697.00	1,704.09	1,707.04
Wald Chi-squared	8.78***	81.10***	7.77***	70.80***
Hosmer and Lemeshow test		p = 0.39		p = 0.40
Lipsitz test		p = 0.34		p = 0.84
Pulkstenis-Robinson test		p = 0.55		p = 0.26

Asterisks and bolding note statistical significance at or below the 10% level; ^⋆^ is < 0.1; ^⋆⋆^ is < 0.05, and ^⋆⋆⋆^ is < 0.01

Standard errors are listed in parentheses. All coefficients have been standardized

Other studies have reached similar conclusions regarding the greater effectiveness of social learning in agriculture compared to formal learning (Hoffman et al. 2015) and independent or institutional learning (Laforge and McLachlan 2018). Social learning has been shown to be a predictor of the adoption of conservation practices in the form of interaction with innovative neighbors (Garbach and Morgan 2017) and attendance at demonstration sites and field days (Singh et al. 2018). Our results mirror findings from Bates and Arbuckle (2017) who looked at farmer preferences for receiving information through face-to-face formats (field days, meetings, or workshops) and non-face-to-face formats (online videos or downloaded publications). The authors found that those who preferred to receive information about nutrient management through face-to-face formats reported using more diverse nitrogen management practices that help reduce nutrient loss (Bates and Arbuckle 2017).

#### Farmer-reported results

**Fig. 5:**
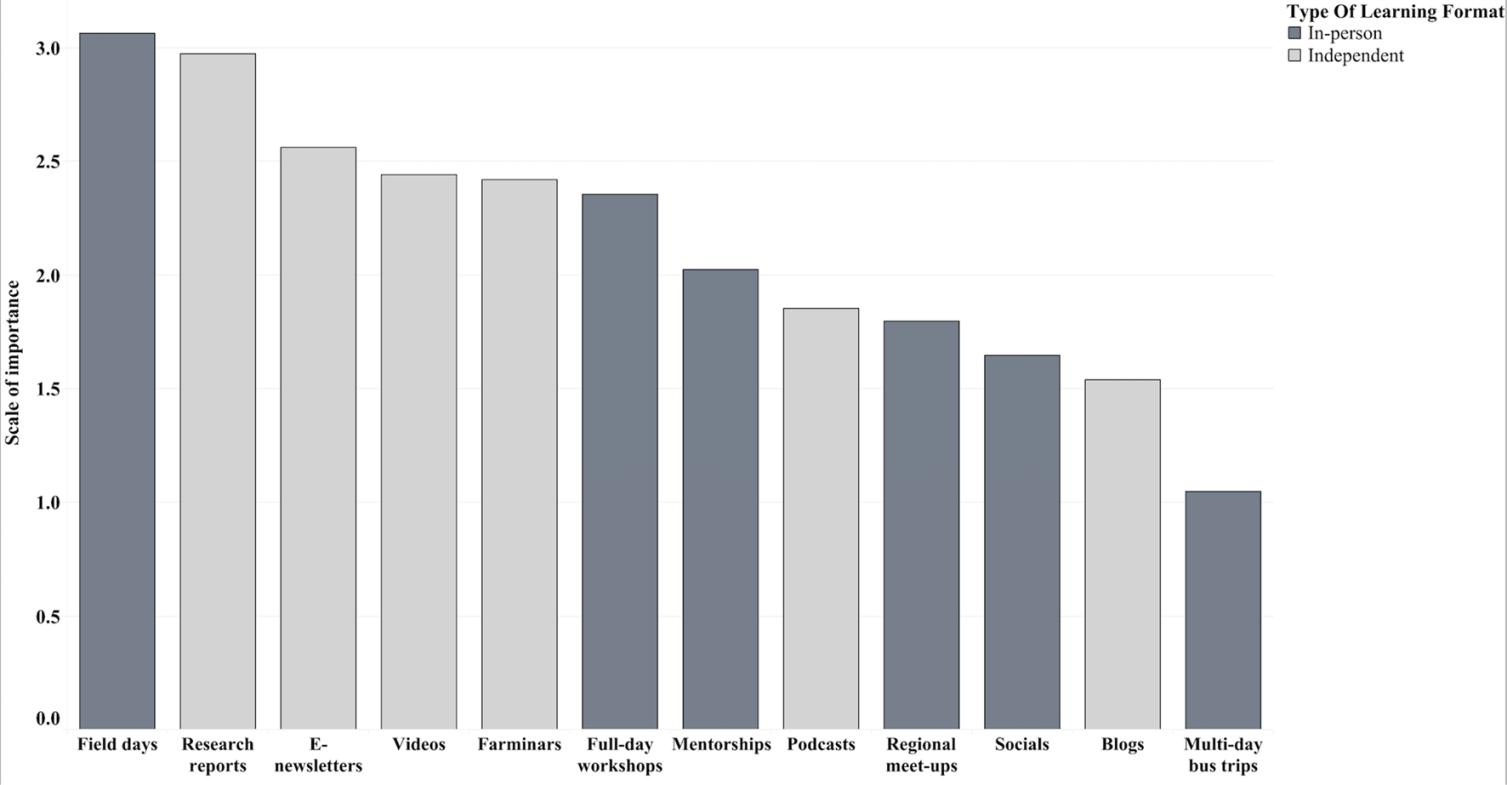
Average ranked importance of PFI learning formats using the member survey data from 2020^a^ (*n* = 410) by type of learning format ^a^Member survey data was not available for this question for the 2013 and 2017 surveys

In terms of preferred ways of learning and participating in the network, farmers reported that they value a range of ways of engaging, including both in-person and independent formats.^4^ In the 2020 member survey, farmers were asked to rate the importance of learning formats offered through the network. The most highly-ranked ways of participating were attending field days followed by reading research reports, reading E-newsletters, watching videos, participating in farminars, and participating in full-day workshops (Fig. 5).

To improve our confidence in the results of the in-person versus independent participation regressions (Models 8–11) and farmer-reported learning preferences in the member survey (Fig. 5), as well as to understand the reasoning behind why farmers prefer one learning format to another, we asked farmers about their learning and participation preferences during in-depth interviews. While farmers reported that they value a combination of in-person and independent learning formats in the survey, during in-depth interviews the majority (68%) of farmers told us that they preferred in-person formats. Farmers said that they use independent sources like research reports and videos for their ease of access, and to stay up to date and connected. However, several key themes emerged around the relatively greater importance of in-person formats that align with existing literature on the benefits of face-to-face interaction for social learning (Bandura 1977; Ban 1981; Rogers 2003): the ability to have side conversations, ask follow-up questions, and to observe results for themselves.

*Side conversations*: Eight of the farmers interviewed discussed the importance of being able to talk with and learn from other farmers during unstructured portions of network events, allowing them to ask follow-up questions and gain a more detailed understanding of the practice. Three illustrative quotes from the interviews are below:

Farmer 5: …some of the things that I do miss since Covid is like, we would go to three, four, five field days in a summer, and the side conversations that we had were just as valuable as the content of the field day.

Farmer 16: …where do you really learn something? It's the breaks or having a beer at the bar afterwards. That’s where you really learn something, in my opinion. [Regarding reading a blog or research report,] I learn something, I learn a concept. But not the details.

Farmer 21: Attending [the field day] live you get some of that community because you can ask questions…what often would happen is you might ask a question and the person who's running the field day will answer it and then you end up having a discussion with the farmer next to you about their answer. You get two farmer's perspectives, not just one...

*Seeing is believing*: A core tenant of farmer-to-farmer learning, as emphasized by Rosset et al. (2011) is “seeing is believing,” which highlights the importance of seeing results with one’s own eyes for the successful diffusion of innovation. This sentiment was echoed by six PFI farmers when explaining the importance of in-person learning. Two of them described this succinctly as:

Farmer 17: The physical presentation of the evidence and getting to see it is just always more impactful than reading about it in a textual format.

Farmer 19: What helps the most is seeing other farmers adopt any new practices and that's where I think that PFI excels is in their field days and showing people how it can be done and making public what their neighbors are doing.

As interviews took place during the first year of the Covid-19 pandemic in the US (July–December 2020), we asked farmers whether their learning and participation preferences were changing due to the newly offered virtual and hybrid formats. Seven farmers expressed appreciation for the flexibility and accessibility of virtual options that they could view on their own time and forgo the travel time and cost, explained by two of them as:

Farmer 2: (Remote field days are) not as good as being in person. But in reality, with taking on the cropping more this year, there is no way I could have taken time to actually drive to all the ones that I’d be interested in. And as much as I really liked doing it in person, the few field days I did attend virtually, it really just took literally the hour that it lasted or whatever. And I could go right back out and work again, and not take the time to drive and come back and whatnot.

Farmer 7: With the Covid this year it was kind of nice because there was a couple field days that I probably wouldn’t have attended due to distance. But because they were on video, I watched them…I could watch it on my time versus when it was happening. I didn’t have to take off of work. It kind of fit my schedule a little better.

One farmer noted the benefits of hybrid participation options that allow for live feedback and questions:

Farmer 15: I think after Covid I’m going to want to go to live events, I’m going to want to go and meet people face-to-face. But I really like the approachability of the Facebook Live in addition to face-to-face, both being held at the same time. I mean, we have the ability to do all that so it's nice to have options.

While nearly all farmers we interviewed appreciated having both in-person and remote options available to them, preferences for face-to-face interaction persisted during the early stages of the Covid-19 pandemic and 72% said that they planned to return to in-person participation when possible. This general sentiment was encompassed by one farmer as:

Farmer 18: I can’t wait to be able to have the in-person stuff again because you still need that interaction with people that listserv and Farminars don’t provide.

These quotes re-iterate the power of in-person learning and help explain the results of the regressions in Table 5 that attest to the greater importance of in-person formats for the adoption of conservation practices. The benefits of and preference for in-person learning likely feeds into the sense of community and relationship building occurring through the network as reported by farmers in the member survey.

## Discussion, limitations, and areas for future research

Because few farmer networks are as large and as long standing as PFI and even fewer collect systematic data from members, we use these unique data to gain greater insight into how the degree of participation in a network is associated with levels of adoption of conservation practices among network participants. We then add to and triangulate the survey data with our qualitative data from in-depth interviews with a subset of member farmers. Our findings support our hypothesis that greater engagement in the PFI network leads to greater use of conservation practices amongst member farmers. Quantitative results show a strong and positive association between participation in the PFI network and the use of conservation practices among members. These results are robust across GLM binomial and ordinal logistic models using pooled data from three years of member surveys. Qualitative interviews reinforce this finding: 76% of farmers told us that participation in the network helped them to transition to sustainable practices, or reinforced their decision to do so, by providing information, resources, encouragement, and confidence building. The effects of participation on adoption are enhanced by participating in the network for a longer period of time, as shown by the positive sign and significance of the interaction between the general participation index and the adoption of conservation practices index. Interviews with farmers suggest this could be due to feedback loops involved with seeing practices used by peer farmers successfully over longer periods of time. For farmers’ participation in the network to be successful, measured as adopting one or more conservation practices as a result of participation, social ties within the network are important, particularly forming relationships. These results add to the literature quantifying the role of formal farmer networks in the adoption of conservation practices and add a nuanced analysis of the role of social networks by measuring the extent of engagement in one well known network in the Midwest.

This research also tests theories of social learning and diffusion of innovation that suggest that in-person, interpersonal channels of communication are a more effective means of changing an individual’s behavior compared to information from impersonal sources. Findings from both our quantitative and qualitative data affirm existing theories of the relatively greater effectiveness of in-person learning, but also point to the complementary nature of the two modes. While PFI farmers appreciated a variety of both in-person and independent ways of engaging in the network, regression results show a stronger relationship between adoption and in-person participation compared to independent participation. In-depth interviews improved our confidence in these regression results by showing that more of the farmers we interviewed (68%) preferred in-person learning formats to support them in their transition to sustainable agriculture. Interviews suggest that this is because PFI farmers are more likely to adopt when they can observe other farmers using a practice, hear about other farmers’ experiences, and ask questions through activities such as field days and workshops.

As researchers continue to search for quantitative models that explain the use of conservation practices in agriculture, our results show that involvement in a formal social network is an important variable for predictive models determining adoption. Our results also show that the type of participation matters, and that when farmers engage in person, they benefit from the direct interaction with peers these formats afford.

However, due to the cross-sectional research design of this study, which it shares with many studies in this literature, we cannot quantitatively prove a causal relationship between the degree of participation in PFI and the adoption of conservation practice because of two main limitations. First, those who join PFI may have certain characteristics that lead them to both participate in a sustainable farming group like PFI *and* to adopt conservation practices. As we saw from the differences between PFI farmers and the general farming population (see Study context), PFI farmers can be considered early adopters and motivated by deeply held values like altruism or other intrinsic motivations. We include covariates in our models that are shown to be associated with adoption in other studies, but due to the limitations of the survey data set, we cannot control for other potential confounding factors that may influence the intensity of both adoption and participation such as environmental ethic, labor availability, free time, and financial resources.

Second, farmers may have adopted a practice before joining PFI or greater adoption may be causing greater participation as opposed to the other way around. While we removed observations that had not been a PFI member for more than one year since taking the survey to ensure that adoption is measured at least one subsequent year since participating in the network, we do not have sufficient data on the same farmers over multiple years to compare each farmers’ change in participation and adoption over time. Once a farmer adopts a conservation practice, the need for information may increase, which is possible given the more knowledge-intensive nature of conservation practices. In this case, adoption may be driving participation as farmers seek additional resources and support. Even if it is the case, this scenario, along with data from farmer interviews, still speaks to the importance of the network as a resource and one that may spur greater or sustained adoption.

Longitudinal studies tracking adoption of a set of farmers before and after network participation, or better yet, experimental studies that randomly facilitate membership in a network and measure any changes in adoption, can help to gain a more precise understanding of whether formal farmer networks are in fact driving the adoption of conservation practices. Still, the effects of network participation are nuanced, with impacts causal studies may not capture. Interview results show that in some cases PFI is a driver of adoption while in others it helps sustain adoption for those that have already started on this path. Both effects are important as the disadoption of conservation practices among farmers is common and can erase their accumulated environmental benefits (Sawadgo and Plastina 2022).

Longitudinal and experimental studies, however, are often more costly, time consuming, and can ask more of already time-constrained farmers. This study uses existing data collected by PFI to contribute to the limited literature aimed at understanding the role of formal farmer-to-farmer networks. While we cannot say from regression results whether farmers who choose to participate in a network may be those already likely to adopt conservation practices, our mixed-methods design allows us to understand causality through multiple modes (Sayer 1992). Across surveys in 2013, 2017, and 2020, 71% of member farmers self-reported that they had adopted conservation practice(s) as a result of participation and during interviews, 76% of a sample of member farmers told us that PFI played a direct role in their transition to sustainable practices, or supported their continued use of these practices. In addition, other research conducted on PFI supports a causal relationship between involvement in PFI and the use of sustainable practices (Bell 2004; Warner 2007; Blesh and Wolf 2014; Carlisle 2016; Blesh and Galt 2017). In a study of Iowa grain farmers by Blesh and Wolf (2014), almost all listed PFI as an important resource for transitioning towards sustainability. The in-depth ethnographic research on PFI from Bell (2004) suggests that the dialogic culture that is a crucial element of PFI is a key way its members were able to transition to more sustainable practices.

Similar to Bell’s (2004) findings, our research shows that building relationships within the network is an important component to adoption as farmers learn and vet ideas through these friendships and feel a sense of community and social support to try different practices. Our interview data show that PFI acts as much more than a channel for the diffusion of information. The network also provides an important source of encouragement and problem solving from like-minded farmers and network leadership, and a means through which to access resources. In the context of formal farmer networks broadly, more research would be useful to understand the ways in which networks encourage farmers to transition to conservation practices and the characteristics of farmer networks that are useful and desirable from farmers’ perspectives. To gain the greatest societal benefit from formal farmer networks, more research is also needed to understand how to encourage greater and sustained participation, how to increase network reach to farmers on the periphery who may benefit most from information exchanged in networks (Granovetter 1973), and how to make networks work better for different types of farmers.

## Conclusion

While our findings are not directly generalizable beyond PFI member farmers, several implications arise for strategies and policies to encourage greater use of conservation practices that may be applicable to farmers in other formal farmer-to-farmer networks in the US and to early adopters of conservation practices broadly. First, formal farmer-to-farmer networks may be a promising way to build and enhance farmers’ technical capacity and provide the peer support necessary to create substantial progress toward sustainable agriculture at a grassroots level. The fact that engagement in PFI (both participation and length of time in the network) and adoption of conservation practices are strongly associated in our analyses, and that member farmers reported during in-depth interviews that PFI helped them to adopt or sustain adoption, suggests that there is a strong synergy between the two, regardless of causality. That early adopters participate frequently in this network means that it is a ripe avenue to support their transition to sustainable agriculture. Supporting these early adopters as role models is likely to catalyze wider-scale change.

Second, to create meaningful change in agriculture, it will be important to understand the most impactful ways of engaging farmers in learning about new practices. This is especially timely to consider given the growing use of remote learning formats brought on by the Covid-19 pandemic. This study finds that while network farmers value both independent and in-person ways of interacting in the network, regression and interview results point to the greater importance of in-person formats in adopting conservation practices. This finding is important as it contrasts other work elevating the importance of self-learning within early adopters compared to later adopters with whom face-to-face exchange is of greater importance (Rogers 2003; Dunn et al. 2016). Thus, networks and other farming organizations may benefit from prioritizing face-to-face programming that encourages relationship-building amongst members. Farmers in this study appreciated the benefits of remote and hybrid ways of learning through the Covid-19 pandemic, yet the strong desire to return to in-person learning formats suggests that remote options are beneficial, when possible, but returning to in-person offerings should be a priority.

The challenge remains that formal farmer networks do not exist everywhere and are limited by a lack of public and private support, and not all farmers have adequate information about networks or the ability to participate. Support is needed to help establish more formal farmer networks focused on conservation and to increase the reach of existing networks to conventional farmers, those in social circles who have not heard about them, and those who may not have the means to participate. We believe that government, land-grant university, and philanthropic investments in, as well as private sector partnerships with, formal farmer networks are a promising way to help farmers transition to more regenerative and resilient practices. Specialized farmer knowledge diffused through social networks is vital for developing farmers’ adaptive capacity to respond to a changing climate, fluctuating markets, and socio-economic instability (Petersen–Rockney et al. 2021). Likewise, Cooperative Extension, embedded in university research, is vital for bringing knowledge from formal institutions to farmers. Creating and disseminating both farmer and formal scientific knowledge, a model unique to formal networks, likely creates synergies far greater than when the two remain separate.

## Footnotes

1 In 2020 and 2017 the survey question read: “Have you changed any of the following as a result of your participation with PFI: conservation practices (reduce inputs, diversifying rotations, cover crops etc.” In 2013 the survey question read: “My participation with PFI helped me improve stewardship on my farm” and noted “Stewardship can be anything you do to leave your farm in better shape for future generations, such as improvements that affect energy use, soil, water, and biodiversity or field conservation.” While the questions are different, we believe that the intent is similar enough to be pooled in one variable for analysis.

2 Two farmers were interviewed together, and their responses are counted as one in the findings because they took turns answering questions.

3 Interview protocol is available on request from the corresponding author.

4 Figure 5 includes only 2020 member survey data. Data was not available for this question for the 2013 and 2017 surveys.
